# Macrophages as gatekeepers of glioma progression

**DOI:** 10.1016/j.omton.2026.201190

**Published:** 2026-04-10

**Authors:** Cliona M. Rooney, Jasia Mahdi

**Affiliations:** 1 Center for Cell and Gene Therapy, Baylor College of Medicine, Texas Children’s Hospital and Houston Methodist Hospital, Houston, TX, USA; 2 Department of Pediatrics, Baylor College of Medicine, Houston, TX, USA; 3 Department of Molecular Virology and Microbiology, Baylor College of Medicine, Houston, TX, USA; 4 Department of Pathology-Immunology, Baylor College of Medicine, Houston, TX, USA; 5 Division of Child Neurology and Developmental Neuroscience, Baylor College of Medicine, Texas Children’s Hospital and Houston Methodist Hospital, Houston, TX, USA

## Main text

A defining feature of glioma progression is the accumulation of immunosuppressive myeloid cells that shape the tumor microenvironment and restrain anti-tumor immunity.^1^ These cells not only support tumor growth but also blunt anti-tumor immune responses, creating a major barrier to effective immunotherapy. In the previous issue of *Molecular Therapy Oncology*, Nazzaro and colleagues investigate whether targeting these suppressive myeloid populations can delay malignant progression in glioma. Using a spontaneous murine glioma model, they show that the macrophage-depleting agent trabectedin reshapes the tumor immune microenvironment, delays tumor growth, and enhances responses to immune checkpoint blockade.

Low-grade gliomas are common central nervous system tumors in adolescents and young adults. These tumors are often slow growing and may remain indolent for prolonged periods of time, although a small fraction may progress and cause significant clinical symptoms, and even death, depending on the location of the tumor. Surgical resection remains the cornerstone of treatment when feasible, while radiation, chemotherapy, and now, molecularly targeted therapies are used for progressive, recurrent, or otherwise higher-risk tumors.^2^ However, chemotherapy and radiation can carry substantial risks, including neurocognitive impairment, endocrine dysfunction, stroke, vasculopathy, and neuropathy, and can increase the risk for secondary malignancy.^3^ Consequently, new therapeutic strategies that treat early disease while minimizing long-term toxicity are urgently needed.

Tumor-associated macrophages (TAMs) are considered major drivers of glioma progression and may constitute up to 50% of the tumor mass.^4^ These cells include both resident microglia and bone-marrow-derived macrophages recruited from the circulation by glioma-derived chemokines. Macrophage abundance correlates with poor prognosis and contributes to tumor progression by suppressing immune effector cells, promoting angiogenesis, and supporting glioma stem cells. Despite their central role in tumor biology, selectively targeting TAMs has been challenging. For example, blockade of colony-stimulating factor (CSF)-1 receptor signaling, an essential pathway for macrophage survival and polarization, produced striking anti-tumor responses in mouse models but failed to improve progression-free survival in patients with glioma in a phase II trial.^5^

Trabectedin, a sequence-specific DNA minor-groove-binding alkylating agent originally isolated from the microbiome of the sea squirt *Ecteinascidia turbinata*, likely evolved as a chemical defense against predators, parasites, and microbes.^6^ The drug induces DNA lesions that are processed by nucleotide excision repair (NER), a process that can convert these lesions into more toxic double-strand breaks requiring homologous recombination for repair.^7^ Tumors with active NER pathways but defective homologous recombination, such as those harboring *BRCA1* or *BRCA2* mutations, are unable to repair these breaks efficiently and are, therefore, particularly sensitive to the drug. Early clinical trials led to its approval for ovarian cancer and advanced soft tissue sarcoma, where disease stabilization was observed in approximately half of patients, although objective response rates remained low.^8^^,^^9^

Several studies have also revealed a selective effect of trabectedin on myeloid cells.^10^ Given the dependence of glioma progression on macrophages, Nazzaro and colleagues examine the effects of trabectedin in an immunocompetent RCAS (Replication-Competent Avian sarcoma-leukosis virus splice-acceptor)-Nestin-TVA (transgenic mice that express the TVA receptor in nestin-positive neural stem cells thought to be the glioma cell of origin) glioma model^11^ that recapitulates spontaneous malignant progression within an M2-biased tumor microenvironment. Intravenous (retro-orbital) injection of trabectedin led to a marked reduction in bone-marrow-derived monocytes and macrophages within the tumor microenvironment, accompanied by a relative increase in microglia and T cells. Similar immune changes were observed systemically in the spleen, whereas myeloid populations in the bone marrow were largely preserved. Trabectedin also induced broader immunological effects, including expansion of intratumoral central and effector memory T cells, increased IL-2 and IL-15 expression, decreased levels of the monocyte chemoattractant C-C motif chemokine ligand (CCL)2, angiogenic vascular endothelial growth factor (VEGF), and reduced regulatory T cell infiltration. Most importantly, trabectedin prolonged survival in tumor-bearing mice and showed enhanced efficacy when combined with anti-programmed death receptor (PD)-1 therapy, although all animals ultimately succumbed to disease. At higher concentrations, the drug also had direct cytotoxic effects on glioma cell lines *in vitro*. The intratumoral effects observed suggest that trabectedin can access the tumor microenvironment following systemic administration, although direct demonstration of blood-brain barrier penetration will require further study.

**Figure 1:**
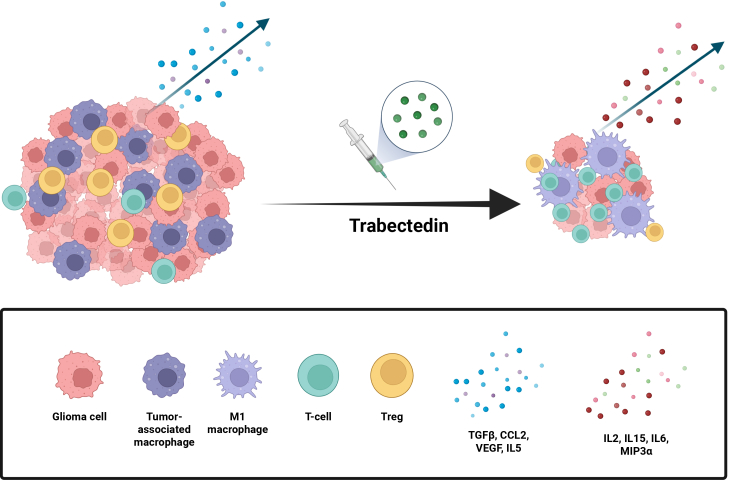
Trabectedin remodels the glioma microenvironment Tumor-associated macrophages accumulate in gliomas, where they suppress T cell responses, promote angiogenesis, and support glioma stem cells, facilitating tumor progression. Trabectedin selectively depletes TAMs through TRAIL-mediated apoptosis, reducing immunosuppressive signaling and permitting increased effector TC infiltration. In the Nazzaro et al. model, this remodeling of the tumor microenvironment delayed glioma progression and enhanced responses to PD-1 (programmmed death receptor-1) blockade.

One component of the macrophage stress response to trabectedin is the induction of TNF-related apoptosis-inducing ligand (TRAIL). Prior studies suggest that the selectivity of trabectedin for M2 macrophages and TAMs may reflect their high expression of the TRAIL receptors DR4 and DR5, which render them particularly susceptible to TRAIL-mediated apoptosis through autocrine and paracrine signaling.^12^ Even at sub-cytotoxic concentrations, trabectedin can inhibit monocyte differentiation into M2-like macrophages (Figure 1). In contrast, T cells and M1 macrophages express lower levels of cytotoxic TRAIL receptors, express decoy receptors that limit TRAIL signaling, and anti-apoptotic molecules such as c-FLICE-like inhibitory protein (FLIP) and Bcl-xL, making them relatively resistant to trabectedin-induced apoptosis.^10^

This intriguing study by Nazzaro et al. supports the concept that suppressive myeloid cells are key drivers of glioma progression and represent a major barrier to effective immunotherapy. By depleting these cells and altering the cytokine milieu, trabectedin appears to transiently relieve myeloid-mediated immunosuppression, allowing T cell responses to emerge. Although trabectedin delayed tumor progression in this model, it did not prevent eventual malignant transformation, and it will be important to determine whether more durable immune remodeling could be achieved through repeated dosing or combination with other immunotherapies. More broadly, this work highlights the possibility that successful immunotherapy for glioma, and likely many other tumors, may require not only activation of T cells but also dismantling the suppressive myeloid networks that act as gatekeepers of tumor progression.

## Declaration of interests

C.M.R.: Founder member Marker Therapeutics, C.M.R. spouse, SAB of Allogene and Tscan; Founder and SAB, March Biosciences.
